# Arteriovenous malformation that caused prolapse of the colon and was treated surgically in an infant: a case report

**DOI:** 10.1186/s40792-020-00824-x

**Published:** 2020-04-08

**Authors:** Miori Kido, Kiyokuni Nakamura, Tsuyoshi Kuwahara, Yoshitomo Yasui, Hideaki Okajima, Nozomu Kurose, Miyuki Kohno

**Affiliations:** 1 Department of Pediatric Surgery, Kanazawa Medical University, Ishikawa, Japan; 2 Department of Pathology and Laboratory Medicine, Kanazawa Medical University, Ishikawa, Japan

**Keywords:** Vascular malformation, Gastrointestinal tract, Pediatrics, Prolapse, Surgery

## Abstract

**Background:**

Various terms have been used to describe vascular lesions in the intestine, including angiodysplasia, arteriovenous malformation, and telangiectasia. Such lesions are common in adults and are typified by angiodysplasia, a type of arteriovenous malformation. In contrast, these lesions are rarely seen in the pediatric population. Angiodysplasia may cause gastrointestinal bleeding, which is sometimes an indication for treatment. Considering the high rate of recurrence after surgical treatment, conservative treatments are mainly chosen. We herein report an extremely rare case of a prolapsed colon due to an arteriovenous malformation successfully treated by resection in a 1-year-old girl. We also highlight the differences between pediatric and adult cases.

**Case presentation:**

A girl developed bloody stools at 7 months of age. She visited another hospital at 1 year of age because of continuing moderate hematochezia and recent onset of rectal prolapse. Colonoscopy showed a protruding lesion located 15 cm from the anal verge, suggesting a submucosal vascular abnormality. Contrast-enhanced computed tomography and magnetic resonance imaging at our hospital revealed the localized lesion with dilated blood vessels in part of the sigmoid colon; no other lesions were present in the gastrointestinal tract. Laparoscopic-assisted sigmoidectomy was performed. A subserosal vascular lesion was visualized and resected using end-to-end anastomosis. Pathologic examination of the 2.2 × 2.7-cm segment revealed several abnormally enlarged and ectatic blood vessels in the submucosa extending into the subserosa. The lesion was diagnosed as an arteriovenous malformation. The patient had a good clinical course without recurrence at the 2-year follow-up.

**Conclusions:**

An arteriovenous malformation in the sigmoid colon may rarely cause intussusception and prolapse of the colon. Complete resection is a radical and potentially effective treatment. Computed tomography and colonoscopy were useful for evaluation of the lesion in the present case.

## Background

Vascular anomalies, which comprise vascular tumors and vascular malformations, can occur in all parts of the body and tend to more commonly affect the skin and soft tissue in children [1, 2]. Various terms have been used to describe vascular lesions in the intestine, including angiodysplasia, arteriovenous malformation (AVM), and telangiectasia [3]. Angiodysplasia is often used to describe AVMs in the intestine of older adults. However, this condition has rarely been reported in the pediatric population [3–11]. Gastrointestinal (GI) bleeding is the main symptom in patients of all ages. Among adults, surgery is reportedly not always the first-line treatment because of the high rate of recurrence. We herein report an extremely rare case of a prolapsed colon due to an AVM that was successfully treated by resection in a 1-year-old girl.

## Case presentation

A previously healthy girl first developed bloody stools at 7 months of age. She then presented to another hospital for continuing moderate hematochezia and rectal prolapse at 1 year of age. She had no any cutaneous lesions or dysmorphosis indicating hereditary syndromes causing vascular lesion, such as Klippel-Trénaunay syndrome, Osler-Weber-Rendu syndrome, blue rubber bleb nevus syndrome, or Proteus syndrome. Colonoscopy performed at that hospital revealed a submucosal lesion with mucosal erosion in the sigmoid colon. Pathologic examination of a biopsy specimen revealed a hematoma. The patient was referred to our hospital for further investigation. A contrast enema revealed no abnormalities of the sigmoid colon or rectum. Colonoscopy showed a protruding lesion located 15 cm from the anal verge, suggesting a submucosal vascular abnormality such as a hemangioma. We planned a second look because the lesion was swollen and covered with erythematous and edematous mucosa, considered to be the result of the rectal prolapse and biopsy.

**Fig. 1:**
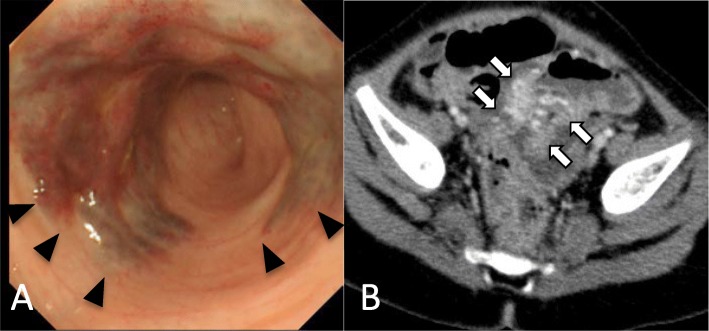
Imaging of the arteriovenous malformation. **a** Colonoscopic view showing the protruding lesion, which was suggestive of a submucosal vascular abnormality. **b** Contrast-enhanced computed tomography image showing dilation of the blood vessels in the lesion, but no hypertrophied feeding artery or draining vein

Repeat colonoscopy performed 2 months later revealed that although the reddening and edema had resolved, the lesion had not changed in size (Fig. 1a). Contrast-enhanced computed tomography (CT) revealed dilation of the blood vessels in the wall of the sigmoid colon, with no other lesions in the GI tract (Fig. 1b).

**Fig. 2:**
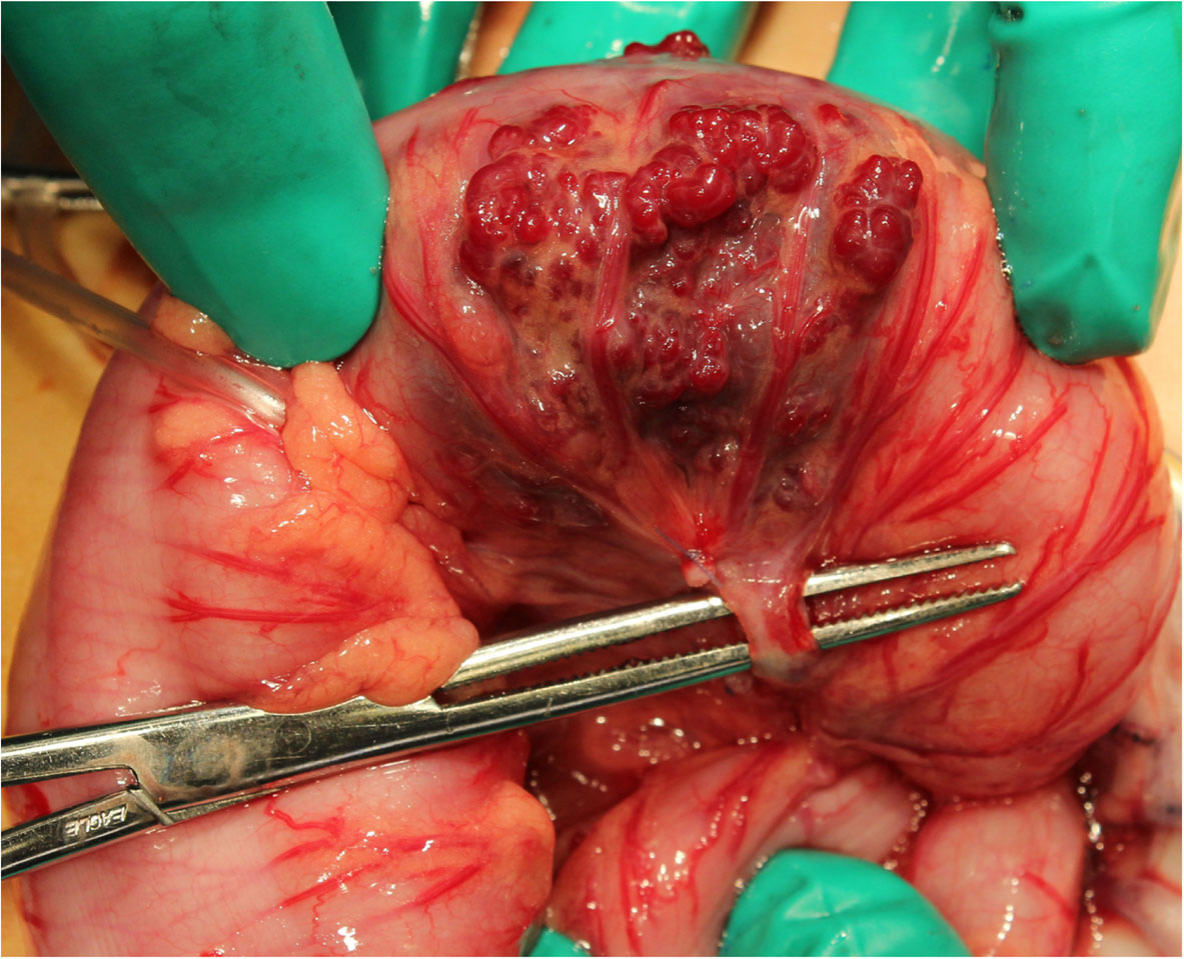
Intraoperative photograph showing the vascular malformation on the serosal surface of the sigmoid colon

Magnetic resonance imaging (MRI) revealed bowel wall thickening in the same area and vascular engorgement with a flow-void appearance. The lesion was thought to be a focal and resectable vascular abnormality; therefore, laparoscopic-assisted sigmoidectomy was performed. An omega-shaped incision was made at the umbilicus, and a multichannel port (EZ accesss; Hakko Medical, Nagano, Japan) was inserted as a platform under direct vision. A 5-mm rigid endoscope was introduced into the EZ Access. A subserosal vascular lesion in the sigmoid colon was visualized (Fig. 2). It had neither thrill nor pulsation.

**Fig. 3:**
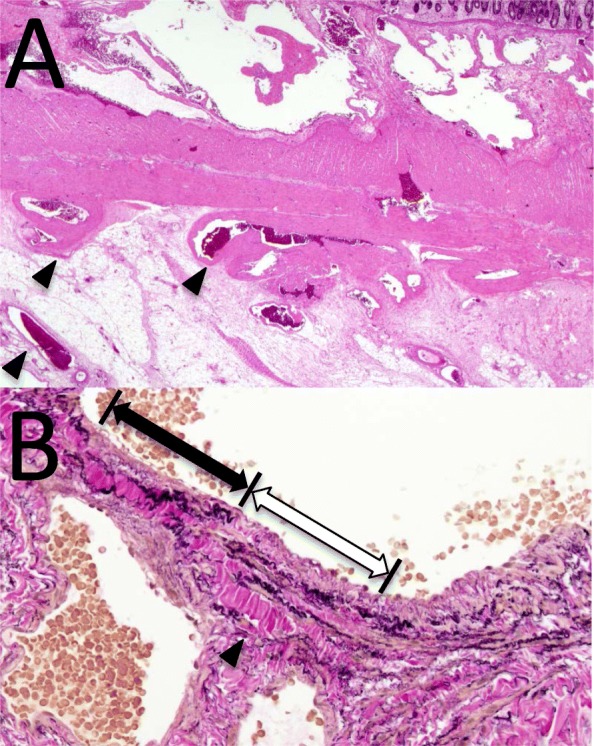
Histological sections. **a** Abnormally enlarged and ectatic vein in the submucosa extending into the subserosa (arrow head). Hematoxylin and eosin stain. **b** Arterial structure with internal elastic lamina and no smooth muscle layer (black arrow) joining a venous vessel with only a smooth muscle layer and no internal elastic lamina (white arrow) certifies the diagnosis of an arteriovenous malformation. The black arrow indicates the adventitia. Elastica van Gieson stain

The lesion was extracted from the abdominal cavity through the umbilical incision and resected using end-to-end anastomosis. Pathologic examination of the 2.2 × 2.7-cm segment revealed several abnormally enlarged and ectatic blood vessels in the submucosa extending into the subserosa. Most of the dilated vessels had characteristics of veins, with thin walls and venous valves, while a few arteries had an elastic lamina and a tangled smooth muscle layer (Fig. 3). The diagnosis was an AVM. No other gastrointestinal lesions were detected by laparoscopic investigation. The patient had a good clinical course without recurrence at the 2-year follow-up.

## Conclusions

In children, vascular anomalies such as infantile hemangiomas are more commonly seen in the skin and soft tissue, while they are rare in the GI tract [1, 2]. Vascular anomalies comprise vascular tumors and vascular malformations [1, 2]. Vascular malformations are congenital morphogenic anomalies of various vessels that present at birth, include dysplastic vessels without cellular proliferation, and have no propensity to regress; in contrast, vascular tumors such as infantile hemangiomas tend to regress spontaneously [1, 2]. They also may occur sporadically, but are often associated with known syndromes such as Klippel-Trénaunay syndrome, Osler-Weber-Rendu syndrome, blue rubber bleb nevus syndrome, and Proteus syndrome. Vascular malformations can be classified by vessel type as capillary, lymphatic, venous, arterial, or complex-combined, such as an AVM [1, 2]. Furthermore, the flow pattern depicted by angiography is used to classify vascular malformations as either slow- or fast-flow [1, 2]. AVMs are classified as high-flow [1, 2]. A previous imaging study showed that the typical CT findings of AVM include highly enhanced lesions with engorged feeding and draining vessels and early opacification of a draining vein in the late arterial or capillary phase [1, 2, 12], while MRI shows a collection of flow-void structures adjacent to the digestive lumen, typically without substantial thickening of the bowel wall [1, 12]. In patients with venous malformations, CT may reveal bowel wall thickening of low attenuation with or without phleboliths, while MRI findings include dilated veins, bowel wall thickening, and associated soft tissue masses that are typically hyperintense on T2-weighted sequences [1, 2, 12]. Although the lesion in our case was pathologically diagnosed as an AVM, the imaging findings were similar to those of a venous malformation, with the absence of an obvious feeding artery on CT and the presence of bowel wall thickening on MRI. This may be because most of the vascular abnormality in this case was composed of veins; thus, too few elastic arteries were present to affect the imaging. Moore et al. [9] further classified AVMs in the GI tract as types I, II, and III based on angiographic characteristics, localization, age of the patient, and family history. Type I lesions appear more commonly in the right colon in adults, type II lesions appear in the small bowel and left colon in pediatric patients, and type III refers to hemangioma-like syndromic lesions [9]. Our patient was considered to have a type II lesion because it was solitary, localized, and apparent on the serosal surface. This case has some differences from adult angiodysplasia. First, pathologic examination in adults reveals dilated, tortuous, thin-walled vessels mainly in the submucosa. In contrast, most pediatric patients, including our patient, had ectatic vessels spreading to the subserosa and some had an elastic lamina; these findings are consistent with AVMs [10, 11].

In addition, intestinal angiodysplasia in the adult population is usually thought to be an acquired degenerative vascular lesion related to aging and associated factors such as aortic stenosis, renal failure, or coagulopathy [4, 6, 7, 10, 11]. The lesions of angiodysplasia may occur in any part of the intestine, most notably in the cecum or ascending colon in adults. They often occur metachronously and multiply. However, as in our case, it has been reported that the localization of angiodysplasia in children often occurs in a solitary location and most commonly in the rectosigmoidal segment [7]. We therefore chose the term “AVM” to describe the lesion in this report. This case suggests that pediatric AVMs in the intestine are different from adult angiodysplasia from the viewpoints of genesis and pathology.

The main symptom in the present case was hematochezia. An AVM might be an important cause of GI bleeding because a previous study showed that double-balloon or capsule endoscopy revealed an AVM in 6.3 to 18.1% of children with occult GI bleeding who underwent examinations by double-balloon endoscopy or capsule endoscopy [13, 14]. AVMs also cause wide-ranging clinical problems such as bowel atresia, perforation [10], and intussusception [15]. To the best of our knowledge, only three cases of AVM-related intussusception have been published, as reported by Lim et al. [15]. In all of these previous cases, the intussusception was located in the small bowel, while the present report describes the first case of an AVM causing colon prolapse due to intussusception of the sigmoid colon. This is because the AVM might have served as a lead point in the sigmoid colon, which is not immobilized.

Because type I lesion in adults are often present in multiple locations and often recur after resection, conservative treatments such as pharmacotherapy, intravascular embolization, and endoscopic argon plasma coagulation are often considered as the initial approach [4, 7]. Several reports have described type I lesion recurrence in children, but all of these recurrences occurred after incomplete resection [5, 8, 9, 12].

Before surgery, correct localization of the lesion is important for complete resection. For the detection of colonic angiodysplasia, CT angiography reportedly has sensitivity, specificity, and positive predictive values of 70%, 100%, and 100%, respectively [6]. Our patient had no other lesions in the GI tract, and the CT findings regarding the boundaries of the lesion were in accordance with the intraoperative findings. CT was useful for deciding the indication and method of surgical treatment.

In summary, an AVM in the sigmoid colon may rarely cause intussusception and prolapse of the colon. Complete resection of an AVM in the GI tract is a radical and potentially effective treatment. Colonoscopy and CT were useful for evaluation of the lesion in the present case.

## Data Availability

The dataset supporting the conclusions of this article is included within the article.

## Abbreviations

GI: Gastrointestinal
CT: Computed tomography
MRI: Magnetic resonance imaging
AVM: Arteriovenous malformation
